# Medium-term clinical results in patients with floating hip injuries

**DOI:** 10.1186/s12893-023-01927-6

**Published:** 2023-02-20

**Authors:** Yun Yang, Chang Zou, Yue Fang, Sujan Shakya

**Affiliations:** Department of Orthopaedics, West China Hospital, Sichuan University, Chengdu, Sichuan People’s Republic of China

**Keywords:** Pelvis, Acetabulum, Femur, Multiple fracture, Floating hip, Operative treatment

## Abstract

**Background:**

The objective of this study was to evaluate the effectiveness of our strategy for managing floating hip injuries.

**Methods:**

From January 2014 and December 2019, all patients with a floating hip underwent surgical treatment in our hospital were included in the retrospective study, with a minimum follow-up of 1 year. All patients were managed according to a standardised strategy. Data on epidemiology, radiography, clinical outcomes and complications were collected and analysed.

**Results:**

Twenty-eight patients were enrolled, with an average age of 45 years. The mean follow-up was 36.9 months. According to the Liebergall classification, Type A floating hip injuries predominated (n = 15, 53.6%). Head and chest injuries were the most common associated injuries. When multiple operative settings were required, we prioritized the fixation of the femur fracture at the first operation. The mean time from injury to definitive femoral surgery was 6.1 days, with most (75%) femoral fractures treated with intramedullary fixation. More than half (54%) of acetabular fractures were treated with a single surgical approach. Pelvic ring fixation included isolated anterior fixation, isolated posterior fixation, combined anterior and posterior fixation, of which isolated anterior fixation was the most common. Postoperative radiographs suggested that the anatomic reduction rates of acetabulum and pelvic ring fractures were 54% and 70%, respectively. According to grading system of Merle d’Aubigne and Postel, 62% of patients achieved satisfactory hip function. Complications included delayed incision healing (7.1%), deep vein thrombosis (10.7%), heterotopic ossification (10.7%), femoral head avascular necrosis (7.1%), post-traumatic osteoarthritis (14.3%), fracture malunion (n = 2, 7.1%) and nonunion (n = 2, 7.1%). In the patients with complications described above, only two patients underwent resurgery.

**Conclusions:**

Although there is no difference in clinical outcomes and complications among different types of floating hip injuries, special attention should be paid to anatomical reduction of the acetabular surface and restoration of the pelvic ring. In addition, the severity of such compound injuries often exceeds that of an isolated injury and often requires specialised multidisciplinary management. Because of no standard guidelines for treatment of such injuries, our experience in the management of such a complex case is to fully assess the complexity of the injury and formulate an appropriate surgical plan based on the principles of damage control orthopaedics.

## Background

The term floating hip is defined as a simultaneous skeletal disruption above and below the hip [1, 2]. The term floating hip was first proposed in 1992 by Liebergall and colleagues to designate a combination of pelvic ring or acetabular fractures and of ipsilateral femoral fractures [3]. Liebergall et al. classified this entity into three types. Type A includes a fracture of the pelvic ring and femur, whereas type B includes a fracture of the acetabulum and femur. Type C is the situation in which both an acetabular and a pelvic fracture are present, and are accompanied by an ipsilateral femoral fracture [3].

This combination of injuries is uncommon, with an incidence of approximately 1/10,000 [1–6]. Studies have found that such injuries are relatively common in jockey race [7]. Like the mechanism of floating knee injuries [8], this kind of injuries is caused by high energy violence. They are often accompanied by traumatic shock, retroperitoneal hematoma, abdominal organ injury and other serious complications, with a high rate of death and disability. Management of this complex pattern of injuries is a serious challenge for trauma orthopedic surgeons and necessitates careful planning and consideration.

Currently, there are relatively few reports on floating hip injuries, limited to a few case reports and clinical cohort studies. Moreover, there is considerable heterogeneity in published studies. In the current state, there is no consensus on the optimal management of such injuries. Therefore, this study retrospectively analyzed the efficacy of floating hip injuries treated at our trauma centre, in order to explore its management strategies.

## Materials and methods

### Subjects

A retrospective evaluation was conducted of patients between January 2014 and December 2019. Inclusion criteria were as following: (i) floating hip; (ii) operative treatment; (iii) regular follow-up. Exclusion criteria included: (i) manifestation of severe osteoporosis, pathological fractures, and previous history of hip or pelvic injuries; (ii) open floating hip; (iii) those patients who were lost in follow-up; (iv) conservative treatment. Epidemiological, clinical, radiological data and complications were collected. Data were collected through an anonymous way because the patients' identifiers such as name and unique identity were erased. The study complied with the Declaration of Helsinki and was approved by the ethics committee of our hospital. All patients provided written informed consent.

### Patient management strategies

1. Early emergency management

According to the Advanced Trauma Life Support (ATLS) protocol, early resuscitation, elimination of fatal causes and saving lives should be the priority. All patients were wearing a pelvic belt when they arrived at the shock unit. These patients were admitted for fluid resuscitation to correct shock. If complicated with abdominal organ rupture or intracranial hemorrhage, laparotomy and craniotomy decompression can be considered as a priority. In addition, evaluation of the soft tissue around the pelvis was mandatory. Open soft tissue injuries or fractures in other areas also needed to be treated as soon as possible. All patients in this group underwent fracture fixation after vital signs were stable. All patients were managed by the same medical team.

(2) Management of fractures

For type A floating hip injuries, the femoral fracture was addressed first to allow effective transosseous traction of the pelvic ring. Reduction and fixation of femoral fracture may be challenging because the effect of the traction bed was diminished by the presence of the concomitant pelvic fracture. In spite of this, closed reduction of the fracture may be attempted first, and open reduction may be considered if necessary. In terms of treatment, attention should be paid to restoring the length of femur and correcting rotation. Secondarily, surgical indications for pelvic fracture were as follows: (1) rotationally unstable but vertically stable fractures with pubic symphysis separation > 2.5 cm or pubic ramus fracture with displacement > 2.0 cm; (2) other rotationally unstable pelvic fracture with significant leg unequal length > 1.5 cm; (3) sacroiliac joint dislocation > 1.0 cm with obvious displacement of sacrum and ilium fractures [9]. For anterior and posterior ring injury of the pelvis, anterior ring injury was fixed by a plate or anterior subcutaneous pelvic fixator (INFIX), while posterior ring injury was fixed by percutaneous sacroiliac screws, or by trans-iliac plate when necessary [10–12].

For type B floating hip injuries, priority fixation of femur fracture is beneficial to reduction of acetabular fracture. Indications for operation included an acetabular fracture with 2 mm or more of displacement, hip instability and incongruence [13]. Acetabular fracture was treated with open reduction and internal fixation (ORIF), combined with percutaneous fixation if necessary. ORIF was recommended for femoral fractures. If the patient had acetabular fracture with major cartilage impaction or femoral head injury and other adverse prognostic factors, the concomitant femoral neck fracture can be treated with hip arthroplasty and same-stage internal fixation of the acetabulum [14].

**Fig. 1:**
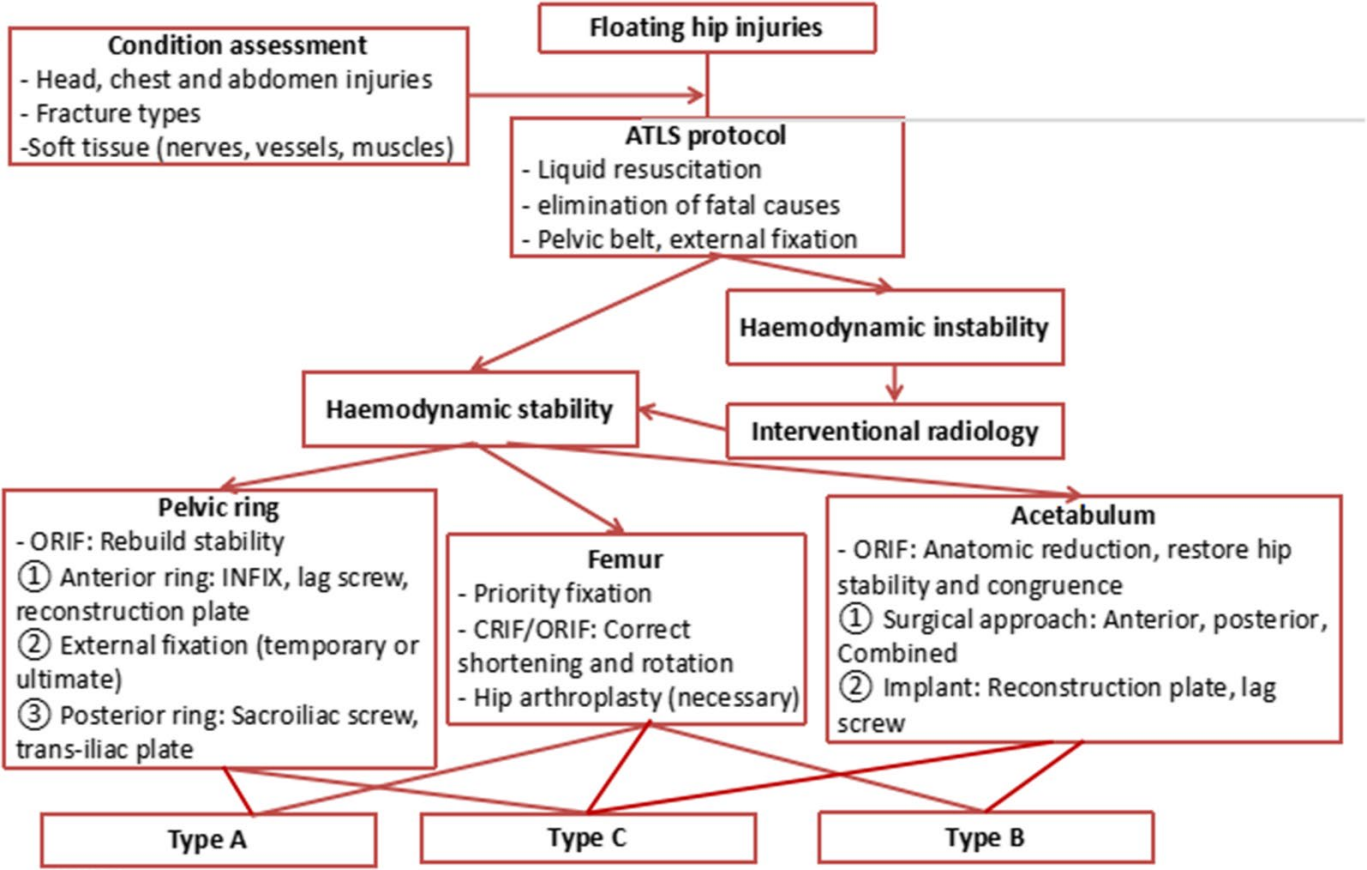
Management strategy for floating hip injuries

In type C floating hip injuries, the initial management can refer to type A and B injuries. The management strategy is described in Fig. 1.

### Evaluation of fracture reduction quality and hip function

According to Matta’s criteria [9], displacement greater than 3 mm on any plain radiographic view indicated a poor reduction. Displacement of 3 mm or less was defined as an imperfect reduction, and an anatomic reduction had 1 mm or less of displacement. To assess the radiological outcomes, Tornetta’s criteria were applied for the pelvic ring [15]. Clinical outcomes at last follow-up postoperatively were scored using the clinical grading system according to Merle d’ Aubigne and Postel [16].

### Statistical analysis

The statistical analysis was conducted using SPSS version 25.0 statistical software (SPSS Inc., Chicago, Illinois, USA). Student’s t test was used for quantitative variables. Categorical variables were analyzed by Pearson’s Chi square test or Fisher’s exact test where appropriate. Value of p below 0.05 was considered as statistically significant.

## Results

### Follow-up data

The shortest follow-up time was 12 months, and the mean follow-up time was 36.9 months.

### Patients and injuries

According to the inclusion and exclusion criteria, a total of 28 patients were included in this study. There were 22 males and 6 females, with an average age of 45 years. The most common mechanism of injury was traffic accident. The average Injury Severity Score (ISS) was 25.1. According to the Liebergall classification [3], more than 50% were type A injuries, which were three times as common as type B injuries and about twice as common as type C injuries. Among the pelvic ring fractures, Tile B fractures were the most common [17]. Based on the Letournel classification [18], the majority of acetabular fractures were complex fractures, with both-column fractures being the most common, accounting for about one-third of all acetabular fractures. For femur fractures, half of them were femoral diaphyseal fractures [19]. Among the associated injuries, chest injuries were the most common, followed by head injuries, and vascular injuries were the rarest. In the above baseline data, there was no significant statistical difference between groups (Table 1).

**Table 1:** The baseline data of the enrolled patients

Variables	Liebergall classification			p value	F/χ^2^
	Type A n (%)	Type B n (%)	Type C n (%)		
Age, years (range)	45.9 (14–80)	40.4 (31–50)	46.3 (31–60)	0.95	0.004
Gender				0.96	0.085
Male (%)	12 (80%)	4 (80%)	6 (75%)		
Female (%)	3 (20%)	1 (20%)	2 (25%)		
Follow-up, months (range)	36.8 (12–72)	48.0 (24–84)	30.0 (12–60)	0.57	0.340
Injury Severity Score (ISS)	21.5 (9–41)	30.0 (13–48)	28.6 (13–48)	0.14	2.337
Mechanism of injury				0.28	5.021
Road traffic accident (%)	10 (66.7%)	2 (40%)	3 (37.5%)		
Fall from height (%)	2 (13.3)	1 (20%)	4 (50%)		
Other (%)	3 (20%)	2 (40%)	1 (12.5%)		
Tile classification [17]				0.27	2.585
Type A (%)	4 (26.65%)	–	0 (0%)		
Type B (%)	7 (46.7%)	–	5 (62.5%)		
Type C (%)	4 (26.65%)	–	3 (37.5%)		
Letournel classification [18]				5.96	0.428
Anterior wall (%)	–	0 (0%)	1 (12.5%)		
Anterior column (%)	–	1 (20%)	0 (40%)		
Posterior wall (%)	–	0 (0%)	2 (25%)		
T-type (%)	–	2 (40%)	1 (12.5%)		
Anterior column and posterior hemi-transverse (%)	–	0 (0%)	1 (12.5%)		
Both-column (%)	–	2 (40%)	2 (25%)		
Transverse and posterior wall (%)	–	0 (0%)	1 (12.5%)		
OTA/AO classification of femoral fracture [19]				0.22	5.760
31 (%)	6 (40%)	2 (40%)	2 (25%)		
32 (%)	7 (46.7%)	1 (20%)	6 (75%)		
33 (%)	2 (13.3%)	2 (40%)	0 (0%)		
Associated injuries				–	–
Head (%)	9 (60%)	4 (80%)	3 (37.5%)		
Chest (%)	12 (80%)	5 (100%)	5 (62.5%)		
Abdomen (%)	4 (26.65%)	1 (20%)	2 (25%)		
Spine (%)	5 (33.3%)	2 (40%)	3 (37.5%)		
Maxillo-facial injuries (%)	5 (33.3%)	2 (40%)	2 (25%)		
Vascular injuries (%)	2 (13.3%)	0 (0%)	1 (12.5%)		
Limb fractures	8 (53.3%)	3 (60%)	2 (25%)		
Total	15 (53.6%)	5 (17.8%)	8 (28.6%)		

Categorical variables were analyzed by Pearson’s Chi square test. Regression analysis was used for continuous variables

### Patient management

All the patients had their pelvic belts temporarily fixed. If the patients still had haemodynamic instability after initial treatment, ATLS therapy should be initiated immediately. Provisional or definitive fixations of unstable fractures were performed as soon as the patients' internal medical were stable. We fixed the femur first, followed by the pelvis or acetabular fracture. The mean time from injury to femoral fixation was 6.1 days. More than 75% of femoral fractures were treated with intramedullary fixation. In type C floating hip injuries, all patients were treated with intramedullary nail fixation of femoral fractures. In spite of floating hip injuries, closed reduction of femur was difficult. However, we performed closed reduction of femur fractures in eight patients (Table 2).

**Table 2:** Surgery-related variables and clinical outcomes

Variables	Liebergall classification			p value	F/χ^2^
	Type A n (%)	Type B n (%)	Type C n (%)		
Injury to femoral surgery time, days (range)	5.3 (2–8)	7.8 (3–11)	6.4 (1–10)	0.253	1.366
Hospital stay, days	15.9	16.4	17.1	0.419	0.639
Closed reduction of femoral fracture (%)	4 (26.65%)	2 (40%)	2 (25%)	0.82	0.397
Definitive femoral fixation				0.18	3.457
Nail (%)	12 (80%)	3 (60%)	8 (100%)		
Locking plate (%)	3 (20%)	2 (40%)	0 (0%)		
Definitive acetabular fixation				0.86	0.747
Anterior approach (%)	–	2 (40%)	3 (37.5%)		
Posterior approach (%)	–	1 (20%)	1 (12.5%)		
Combined approaches (%)	–	2 (40%)	3 (37.5%)		
Total hip arthroplasty (%)	–	0 (0%)	0 (0%)		
Non-surgical treatment (%)	–	0 (0%)	1 (12.5%)		
Definitive pelvic ring fixation				0.624	4.387
Anterior ring only	0 (0%)		0 (0%)		
Symphyseal plate (%)	7 (46.7%)	–	2 (25%)		
INFIX (%)	3 (20%)	–	3 (37.5%)		
External fixator	1 (6.66%)		0 (0%)		
Posterior ring only					
Sacroiliac screw (%)	1 (6.66%)	–	0 (0%)		
Anterior and posterior rings		–			
INFIX + sacroiliac screw (%)	2 (13.3%)	–	1 (12.5%)		
External fixator + sacroiliac screw (%)	0 (0%)	–	1 (12.5%)		
Symphyseal plate + sacroiliac screw (%)	1 (6.66%)		1 (12.5%)		
Quality of reduction					
Pelvic ring [15]				0.579	1.969
Excellent (%)	11 (73.3%)	–	5 (62.5%)		
Good (%)	2 (13.3%)	–	1 (12.5%)		
Fair (%)	1 (6.7%)	–	0 (0%)		
Poor (%)	1 (6.7%)	–	2 (25%)		
Acetabulum [9]				0.940	0.124
Anatomical (%)	–	3 (60%)	4 (50%)		
Imperfect (%)	–	1 (20%)	2 (25%)		
Poor (%)	–	1 (20%)	2 (25%)		
Assessment of hip function [16]				0.513	2.297
Excellent (%)	–	2 (40%)	3 (37.5%)		
Good (%)	–	1 (20%)	2 (25%)		
Fair (%)	–	0 (0%)	2 (25%)		
Poor (%)	–	2 (40%)	1 (12.5%)		
Total	15(53.6%)	5(17.8%)	8(28.6%)		

Categorical variables were analyzed by Pearson’s Chi square test. Regression analysis was used for continuous variables

All acetabular fractures were treated with ORIF except for one patient whose family refused further surgery due to deterioration of his condition. One third of patients had a anterior approach, the same as those who had a combined approach. None of the patients underwent primary total hip arthroplasty (THA) (Table 2).

**Fig. 2:**
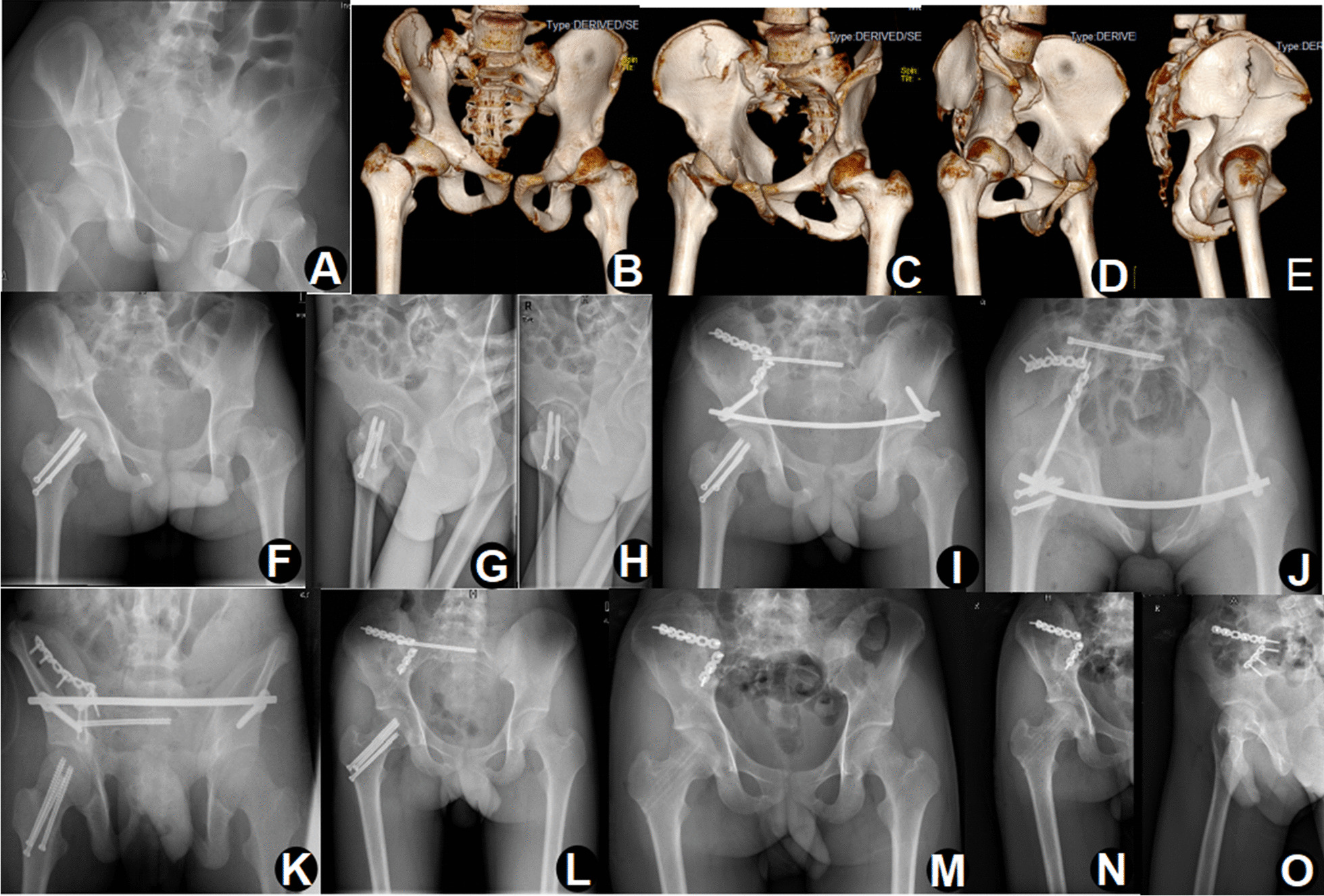
A 16-year-old male treated with damage control orthopaedics. Initial radiographs (**A**) and three-dimensional computed tomography images (**B**–**E**) showing a type C floating hip. Closed reduction and internal fixation of femoral neck fracture on the third day after admission (**F**–**H**). Percutaneous sacroiliac screw fixation of the posterior ring, INFIX fixation of the anterior ring, and anterior fixation of the acetabular fracture by the lateral window of the IL approach on the tenth day (**I**–**K**). The removal of INFIX 1 year after surgery (**L**). The removal of cannulated screws and sacroiliac screw 1.5 years after surgery (**M**–**O**)

The treatment of the pelvic ring fractures included isolated anterior fixation, isolated posterior fixation, combined anterior and posterior fixation. All patients underwent surgical stabilization for at least one component of pelvic ring disruption. An isolated anterior ring fixation was used in nearly 75% of patients. With the rise of INFIX, we used INFIX to fix the anterior pelvic ring in nine patients (Fig. 2). All posterior ring fractures were fixed with sacroiliac screws under navigation (Table 2).

### Radiological and clinical outcomes

According to the Tornetta’s criteria [15], anatomical reduction was achieved in 16 patients. The overall excellent and good rate reached 83%. The good rate of reduction quality of type A floating hip injuries was higher than that of type C floating hip injuries. Application of the Matta’s criteria [9] to the 13 acetabular fractures indicated that reduction was imperfect or excellent in 10 (77%) patients (Table 2). Based on the hip function score criteria [16], 62% of patients achieved satisfactory hip function (Table 2).

### Complications

The complications seen in these patients included early and late complications. These complications were detailed in Table 3. Early complications included delayed incision healing (n = 2, 7.1%) and deep vein thrombosis (DVT) (n = 3, 10.7%). Wound-healing problems were healed by prolonged antibiotic use and infrared lamps. Patients with DVT confirmed by duplex ultrasonography were treated with low molecular weight heparin while bedridden. There were no episodes of clinically diagnosed pulmonary embolism.

**Table 3:** Complications

Complications	Liebergall classification			p value
	Type A n (%)	Type B n (%)	Type C n (%)	
Early				
Delayed healing (%)	1 (6.66%)	0 (0%)	1 (12.5%)	0.69
Deep vein thrombosis (%)	2 (13.3%)	1 (20%)	0 (0%)	0.47
Late				
Heterotopic ossification (%)	0 (0%)	1 (20%)	2 (25%)	0.14
Post-traumatic osteoarthritis (%)	1 (6.66%)	1 (20%)	2 (25%)	0.45
Avascular necrosis (%)	0 (0%)	1 (20%)	1 (12.5%)	0.25
Non-union (%)	1 (6.66%)	1 (20%)	0 (0%)	0.39
Femur (%)	1 (6.66%)	1 (20%)	0 (0%)	0.39
Pelvic ring (%)	0 (0%)	0 (0%)	0 (0%)	–
Acetabulum (%)	0 (0%)	0 (0%)	0 (0%)	–
Malunion (%)	1 (6.66%)	0 (0%)	1 (12.5%)	0.69
Femur (%)	0 (0%)	0 (0%)	0 (0%)	–
Pelvic ring (%)	1 (6.66%)	0 (0%)	1 (12.5%)	0.69
Acetabulum (%)	0 (0%)	0 (0%)	0 (0%)	–
Total	15 (53.6%)	5 (17.8%)	8 (28.6%)	28(100%)

**Fig. 3:**
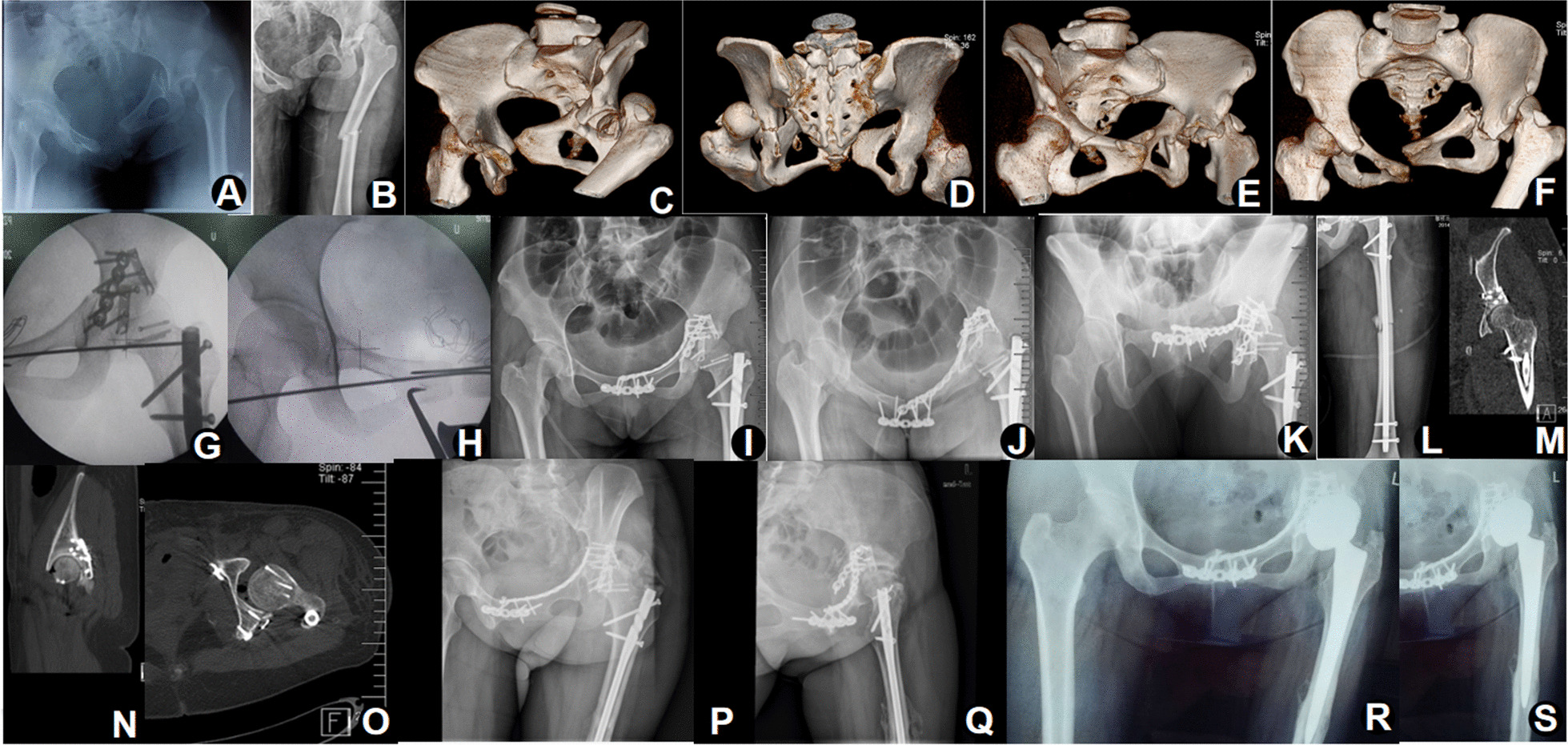
A 27-year-old female treated with injury control theory. Preoperative images suggested type C floating hip injury revealing a type C floating hip (**A**–**F**). An antegrade intramedullary fixation of femoral shaft fracture, followed by posterior fixation of the femoral head and posterior wall of the acetabulum and finally anterior fixation of the anterior ring and anterior column of the acetabulum on the seventh day after the injury (**G**, **H**). Immediate postoperative radiographs showing satisfactory reduction of all fractures (**I**–**L**). Postoperative CT suggesting anatomic reduction of acetabular fracture (**M**–**O**). Radiographs at 2 years of follow-up revealing severe post-traumatic osteoarthritis of the left hip with heterotopic ossification (**P**, **Q**), which was managed by implant removal and left THA at 3.5 years after the initial surgery (**R**), and the radiograph of 1 year after arthroplasty indicating no loosening of the prosthesis (**S**)

Late complications included heterotopic ossification (n = 3, 10.7%), post-traumatic osteoarthritis (n = 4, 14.3%), avascular necrosis of the femoral head (n = 2, 7.1%), fracture malunion (n = 2, 7.1%) and nonunion (n = 2, 7.1%). Of the three patients with heterotopic ossification, two patients chose conservative treatment, and the remaining patient chose THA due to severe clinical symptoms and concomitant post-traumatic osteoarthritis (Fig. 3). Among the four patients with post-traumatic osteoarthritis, except the one mentioned above who received THA, the other three patients had mild symptoms of osteoarthritis, which were relieved to varying degrees by oral drugs. One of the two patients with nonunion of femur fracture, who was asymptomatic at the last follow-up and had poor financial conditions, refused further surgery. The other had revision surgery, where bone grafts and implants were replaced to heal the fracture. Two patients with malunion of pelvic ring fracture chose conservative treatment because their symptoms were mild and did not affect daily walking (Table 3).

## Discussion

Floating hip injuries render hip joint unstable both proximally and distally, which can be either extra-articular or intra-articular. These types of injuries usually result from high-energy trauma and are combined with head, chest, abdomen or other injuries [1, 2, 6, 20–23]. Such injuries are relatively rare. The management of the combination of injuries is a thorny issue due to the low incidence and the lack of literature on their management [23, 24].

Damage control orthopaedics offers a step-wise approach to the management of patients with multiple injuries [25]. In applying this approach, several deep issues have to be considered. First, it is necessary to understand the type of floating hip injury and the mechanism of injury. Second, the timing of the decisive surgery is determined. Further, the sequence of fracture fixation is determined. Finally, the appropriate surgical approach should be developed.

### Injury mechanism and fracture patterns

Liebergall et al. [2] reported two main patterns of floating hip injury based on the mechanism of injury: the posterior type injury and the central type injury. The former was found mostly among the passengers in the front seat, which was mainly caused by a blow force (“dashboard injury”) on the knee through the femur transferred to the posterior elements of the acetabulum. The latter was found mostly among patients that fall from height or pedestrians who were struck by a car, which was mainly caused by a lateral blow force on the trochanteric region (“lateral impaction injury”) through the femoral head transferred to the hip. In our study, the most common injury mechanism was road traffic accident, followed by fall from height. According to the classification of Liebergall [3], floating hip injuries were divided into three types based on different combinations: type A (pelvic ring and ipsilateral femoral fractures), type B (acetabular and ipsilateral femoral fractures) and type C (all three fractures present). Type A floating hip injury accounts for about 65%.

### Surgical timing

The time from injury to the first surgery can range from a few hours to several days. Burd et al. retrospectively reviewed 57 patients of floating hip and reported the mean time was 87 h from injury to first operative stabilization [6]. Liebergall et al. reported that acetabular fractures were operated on 3–5 days after injury, while the fixation of femoral fractures were recommended to be completed within 24 h after injury [2]. Anyway, attempts to form a protocol for surgical timing should be subjected to damage control orthopaedics that attaches importance to the physiological state and associated injuries [2, 6, 26, 27]. If the patient was hemodynamically stable and the surgeon was familiar with the anatomy of the fracture site, one-stage fixation may be considered for all fractures. In our report, the mean time from injury to definitive femoral surgery was 6.1 days, longer than the time reported in some literature [6, 28]. The main reason was that most patients were referred from other hospitals. Because the injury was too severe to be transported, life support was required at a local hospital, which delayed definitive treatment of the fracture.

### Operation order

There has been a disparity in the sequence of fracture fixation. While Kregor et al. [23] suggested the fixation of acetabular fractures should be a priority, some suggested that the fixation of the femur fracture should precede definitive management of pelvic and acetabular fractures [2, 5, 6]. In our cases, we prioritized femoral fractures as stabilization of the femur fracture could facilitate exposure, traction, reduction and fixation of the pelvic or acetabular fracture. Some scholars recommended the ORIF and bone strut allograft technique for Multifragmentary segmental femoral shaft fractures in floating hip injuries [29].

### Surgical approach

Appropriate surgical approach should meet the requirements of intraoperative exposure and operation while minimizing surgical trauma. For the posterior type of floating hip, it is possible to address the acetabular and femoral fractures through the Kocher-Langenbeck (KL) approach. Antegrade intramedullary nailing can be inserted through a small percutaneous incision in the lateral position and the incision can easily be incorporated into the KL approach if necessary. In the case of a femoral shaft or distal femoral fracture, retrograde intramedullary nailing can be used to avoid interference with the proximal incisions that may be required. Since the central type of floating hip do not involve the posterior elements of the acetabulum in most cases, acetabular fractures can be fixed by the anterior approach, while the femoral fracture is fixed through another lateral incision. Both fractures in this combination could also be simultaneously addressed by extensile ilio-femoral approach if the patient is able to tolerate prolonged surgery. In our cases, the combined approach was used in only 5 patients. Acetabular fracture fixation was accomplished in most patients through a single surgical approach, which minimized surgical trauma without compromising reduction quality.

### Complications

Currently, the complications of floating hip injuries reported in literature included DVT, avascular necrosis of the femoral head, post-traumatic osteoarthritis of the hip, heterotopic ossification (HO), traumatic sciatic nerve palsy, among which traumatic sciatic nerve palsy was the most severe [1, 6]. In our study, two patients underwent resurgery for severe post-traumatic osteoarthritis and femoral fracture nonunion, respectively. After THA and femur revision surgery respectively, both patients achieved satisfactory function and were able to meet work and basic life needs.

This study has several limitations. First, the retrospective design can only provide a low level of evidence. Second, data were collected from single Level-1 trauma center and may affect their representativeness and consistency. Third, we excluded open floating hip injuries, which made the complexity of such serious injuries relatively simple and may interfere with the accuracy of clinical results. Finally, the limitation of our work was the heterogeneity and the small size of the included cases. Therefore, further research could expand the coverage and diversity of samples and increase layers of research design.

## Conclusions

In conclusion, floating hip injury is a devastating injury. Attention should be paid to the anatomical reduction of the acetabular articular surface and the recovery of the pelvic ring in the treatment of such injuries. Due to the severity and complexity of floating hip injury, multidisciplinary cooperation is often required. The general principle is to fully assess the complexity of injury and perform definitive surgery as early as possible on the basis of damage control orthopaedics.

## Data Availability

The datasets generated during the current study are not publicly available due to the limitations of hospital regulations but are available from the corresponding author on reasonable request.

## Abbreviations

ATLS: Advanced Trauma Life Support
ORIF: Open reduction and internal fixation
ISS: Injury Severity Score
THA: Total hip arthroplasty
DVT: Deep vein thrombosis
KL: Kocher-Langenbeck
HO: Heterotopic ossification
